# Supplementary material for: Chromothripsis during telomere crisis is independent of NHEJ, and consistent with a replicative origin
Source: Genome Res. 2019 May;29(5):737–49. doi: 10.1101/gr.240705.118 (PMC6499312; doi:10.1101/gr.240705.118)
Supplement: Supplemental Material [file supp_gr.240705.118_Supplemental_file_1.zip › contigs/annotated_contigs/DB114/contig.2.DB114_length_267_mean_cov_16.1161048689.docx]

**DB114_length_267_mean_cov_16.1161048689**

```
ATGTTGGGAAGTCGGGTAAAAAACTGACCAGCAAACAACAAATCACTGCCTGACATCCCCCCA|C|CCCCCCCCCCCCCCCCCCCCCCA
 >chr11:675850-675913 + E=1e-26                                  >chr2:33141456-3314165
CCCCCCCCCCCCCCCCCCCCCCCACCCCCCCCCCCCCCCCCCCCCCCCCCCCCCCCCCCCACCCCCCCCCCCCCCCCCCCCCCCCCCCC
3 - E=3e-74
CACCCCCCCCCCCCCCCCCCCCCCCCACCCCCCCCCCCCCCCCCCCCCCCACCCCCCCCCCCCCCCCCCCCCCTCCACCCCCCCC|AAC

TCC
```
